# Changes in gut viral and bacterial species correlate with altered 1,2-diacylglyceride levels and structure in the prefrontal cortex in a depression-like non-human primate model

**DOI:** 10.1038/s41398-022-01836-x

**Published:** 2022-02-22

**Authors:** Jing Wu, Tingjia Chai, Hanping Zhang, Yu Huang, Seth W. Perry, Yifan Li, Jiajia Duan, Xunmin Tan, Xi Hu, Yiyun Liu, Juncai Pu, Haiyang Wang, Jinlin Song, Xin Jin, Ping Ji, Peng Zheng, Peng Xie

**Affiliations:** 1 Department of Neurology, The First Affiliated Hospital of Chongqing Medical University, Chongqing, 400016 China; 2 NHC Key Laboratory of Diagnosis and Treatment on Brain Functional Diseases, The First Affiliated Hospital of Chongqing Medical University, Chongqing, 400016 China; 3 The M.O.E. Key Laboratory of Laboratory Medical Diagnostics, the College of Laboratory Medicine, Chongqing Medical University, Chongqing, 400016 China; 4 College of Biomedical Engineering, Chongqing Medical University, Chongqing, 400016 China; 5 Department of Psychiatry and Behavioral Sciences, College of Medicine, State University of New York (SUNY) Upstate Medical University, Syracuse, New York USA; 6 Department of Neuroscience & Physiology, College of Medicine, SUNY Upstate Medical University, Syracuse, New York USA; 7 Chongqing Key Laboratory of Oral Diseases and Biomedical Sciences, Stomatological Hospital of Chongqing Medical University, Chongqing, 401147 China; 8 Key Laboratory of Psychoseomadsy, Stomatological Hospital of Chongqing Medical University, Chongqing, China

**Keywords:** Human behaviour, Diagnostic markers, Depression

## Abstract

Major depressive disorder (MDD) is a debilitating mental disease, but its underlying molecular mechanisms remain obscure. Our previously established model of naturally occurring depression-like (DL) behaviors in *Macaca fascicularis*, which is characterized by microbiota-gut–brain (MGB) axis disturbances, can be used to interrogate how a disturbed gut ecosystem may impact the molecular pathology of MDD. Here, gut metagenomics were used to characterize how gut virus and bacterial species, and associated metabolites, change in depression-like monkey model. We identified a panel of 33 gut virus and 14 bacterial species that could discriminate the depression-like from control macaques. In addition, using lipidomic analyses of central and peripheral samples obtained from these animals, we found that the DL macaque were characterized by alterations in the relative abundance, carbon-chain length, and unsaturation degree of 1,2-diacylglyceride (DG) in the prefrontal cortex (PFC), in a brain region-specific manner. In addition, lipid-reaction analysis identified more active and inactive lipid pathways in PFC than in amygdala or hippocampus, with DG being a key nodal player in these lipid pathways. Significantly, co-occurrence network analysis showed that the DG levels may be relevant to the onset of negative emotions behaviors in PFC. Together our findings suggest that altered DG levels and structure in the PFC are hallmarks of the DL macaque, thus providing a new framework for understanding the gut microbiome’s role in depression.

## Introduction

Major depressive disorder (MDD) is a serious mental disorder that imposes a substantial burden on families and society worldwide [1]. Currently, the underlying molecular basis of MDD remains uncertain. In addition, only a subset of patients with MDD are effectively treated by the currently available antidepressant drugs, which have been developed based on the existing theories for the molecular basis of MDD [2, 3]. Therefore, identification of new molecular mechanisms and more effective therapies for MDD are required.

Emerging evidence suggests that the gut microbiome can modulate brain function and behaviors through the microbiota-gut–brain axis (MGB), and plays a vital role in the pathogenesis of various mental disorders such as autism, bipolar disorder, and schizophrenia [4–7]. Recently, we and others found that patients with MDD had disturbed gut microbiomes, but the underlying mechanisms by which the gut microbiome may contribute to the pathogenesis of MDD are unclear. Previously, we established and reported a non-human primate (NHP) model of naturally occurring depression-like behaviors, which developed under conditions of chronic social dominance stress [8, 9]. This unique animal model replicates many characteristics of depression onset and behaviors in humans. This primate DL model was also characterized by MGB axis disturbances, thus providing an ideal model to investigate the gut ecosystem’s roles in depression development.

Previously, integrated metabolomic and metagenomic analyses in these DL macaques found that their altered gut microbiota were associated with brain-region specific glycerophospholipid metabolism changes the prefrontal cortex (PFC), amygdala (AMY), and hippocampus (HIP) [8]. Similar alterations were also found in the PFCs of germ-free mice that received fecal microbial transplants (FMTs) from MDD subjects [10]. Gut microbiota can influence MGB lipid and fatty acid production and metabolism, and lipid peroxidation and signaling pathway activation [11]. However, further studies are needed to better clarify the molecular interrelationships and downstream functional consequences of these changes on MDD pathology.

Lipids are a group of distinct biological molecules which make up over 50% of the brain’s dry mass, and are vital participants in fundamental neurobiological processes including neuronal membrane synthesis, and vesicular and synaptic transmission and trafficking. There are major subclasses of lipids such as fatty acids; glycerolipids such as mono-, di-, and tri-acylglycerol (MG, DG, and TG, respectively); and glycerophospholipids such as phosphatidylcholine (PC), phosphatidic acid (PA), and phosphatidylglycerol (PG). In the brain, with various enzymatic reaction systems, lipids achieve rapid transformations across subclasses, and disrupted lipid transformations (and thus equilibrium) can manifest as a variety of neurological diseases [12, 13]. Moreover, the structures and dynamic activities of neural cells’ membranes are profoundly sensitive to lipid structure, including acyl carbon chain length and unsaturation [14]. For neurons and glia, and their vesicles, changes in membrane permeability and fluidity, as regulated by lipid composition and structure, help control synaptic excitability and transmission, and the stabilization of ion channels [15, 16]. Short-chained and saturated lipids generate a more tightly packed and compressed cell membrane whilst, in contrast, longer-chained and unsaturated lipids occupy more space and are more mobile in membranes.

For these reasons, here we used shotgun metagenomic analysis of fecal samples, and mass spectrometry (MS) lipidomic analysis of brain regions (PFC, AMY, and HIP) and plasma, from 12 female DL macaques (*Macaca. fascicularis*) and healthy controls (HC). With these well-established bioinformatic approaches, the differential gut virus, bacteria, and brain lipids between the DL and HC macaques were identified. Based on metabolic reactions among lipids, we identified the involved pathways and predicted the active or inactive lipid reaction pathways in key brain regions involved in MDD pathology. Finally, we integrated these multi-level omics findings by network analysis to connect and correlate the altered virus and bacteria with the observed DL behaviors, to further uncover how these disturbed signatures may modulate the host’s metabolism and behavioral function.

## Materials and methods

### Ethics Statements

Detailed ethics approvals were shown in Declaration. In brief, this study was performed in strict accordance with the recommendations in the “Guide for the Care and Use of Laboratory Animals” of the Institute of Neuroscience at Chongqing Medical University (#20100031). The sample sizes (n = 6/every group) were set following the nc3r recommendations (https://www.nc3rs.org.uk/) by using the minimum number of NHP, as well as previous studies involving NHP brains [17, 18], relatively smaller than our previous works on behavior and biochemical measures in *M. fascicularis* [19]. Macaques were housed in an environmentally controlled facility (22 ± 1 °C temperature; 50 ± 5% relative humidity; and 12 h light/12 h dark cycle with lights on at 7:00 AM), and were fed at same time(7:30, 12:00, 15:00).

### Behavioral observation and tissue preparation

We identified *M. fascicularis* animals with naturally occurring DL behaviors as described previously [8, 19, 20]. Briefly, we surveyed the populations from 20 enclosures, and identified 6 typical DL and 6 HC macaques. All 12 identified macaques were adult female and disease-negative by veterinary examination. The DL behaviors huddle, sit alone, locomotion, and amicable were observed and identified via “free enclosures tests” [8, 20]; the behavior communication was evaluated and identified by “social interaction test” [8]. Free enclosures test was conducted in macaque native enclosures. Experimenters quietly record videos without disturbing the subjects. Social interaction test was conducted in two adjacent single cages (transparent partitions) in which a male macaque and a female macaque were placed separately. The female macaque would be attracted or threatened by the male one to climb in different place in its cage. Experimenters quietly record videos without disturbing the subjects.

All behavioral observations were videotaped and analyzed by NOLDUS Observer XT software (version 10.0, Noldus Information Tech Technology, Leesburg, PA). The experimenter would identify the target behaviors and record the spending time. Behaviors can be briefly described as following: huddle, solitary huddling and heading down under shoulder; sit alone, sitting far away from others; amicable, receiving or providing amicable grooming; ingestion, eating at public trough; locomotion, walking, running or climbing; communication, in social interaction test, the female macaque cross the center line and to contact the male one [8, 19, 20]. Based on the similarity with human emotions, behaviors huddle and sit alone were identified as negative emotions, behaviors locomotion, amicable and communication were identified as positive emotions. The detailed behavioral results have been published previously [8] and reprinted in supplementary table 1 to aid readability.

Following euthanization, whole brains were isolated and immediately frozen in liquid nitrogen, and cut into 5 mm slices along the coronal plane [21, 22]. The PFC, HIP, and AMY were dissected according to a macaque brain atlas, then stored at −80 °C until use.

### Metagenomic analysis of fecal samples

The metagenomic libraries were prepared and sequenced according to our previous protocols [23, 24]. Briefly, we extracted microbial DNA from the 6 DL and 6 HC macaque fecal collections using the E.Z.N.A. Stool DNA Kit (Omega Bio-tek, Norcross, GA, USA). The DNA extract was sheared using Covaris M220, aimed insert size was about 300 bp. A paired-end library was constructed using TruSeqTM DNA Sample Prep Kit (Illumina). Then, the amplicons library was paired-end sequenced on an Illumina HiSeq X platform [25]. Threshold exclusion in sequence quality control and genome assembly was performed as we described previously [23, 24]. Reads were aligned to the macaque (*Macaca fascicularis*) genome by BWA (http://bio-bwa.sourceforge.net), and any hit associated with the reads and their mated reads were removed. Metagenomics data were assembled using MEGAHIT [26] (https://github.com/voutcn/megahit), contigs with the length being or over 300 bp were selected as the final assembling result. All predicted genes with a 95 % sequence identity (90% coverage) were clustered using CD-HIT [27] (http://www.bioinformatics.org/cd-hit/). Reads after quality control were mapped to the representative sequences with 95% identity using SOAPaligner [28] (http://soap.genomics.org.cn/). Here, prior to construction of gene sets, we initially removed the host sequences with high homology to the macaca genome from microbial metagenomes. Based on the NCBI NR database, we annotated gene sets for bacteria, fungi, viruses, protozoa, and archaea using Diamond (Version 0.8.35). Based on a unified database, each gene is assigned to the highest scoring taxonomy, which facilitates simultaneous assessment of these microbial species in the gut ecosystem of depression-like macaca [29]. The differential bacterial species and gut viruses between the two groups were identified using Linear discriminant analysis (LDA) Effect Size (LEfSe) with an LDA score >2.0 [30].

### LC-MS for lipidomics

Details of the lipidomic methods were based on Liquid chromatography-mass spectrometry (LC-MS), similar to our previously published protocols [8, 24]. Brain tissue samples were prepared by homogenization, dissociation, and centrifugation, and plasma samples were collected and centrifuged twice [23]. To ensure the reliability of our lipidomic protocol, mixed brain tissue samples were divided into three portions and used as quality control (QC). In detail, for all the brain tissues, we first weighed 30 mg (±2 mg) into the EP tube, then added 800 μl methanol/water mixture (1:1, v/v) and small steel balls. For plasma, we used 100 μl per sample. Next, tissue homogenate was performed using an automatic tissue grinder (Shanghai Jingxin Co., Ltd), the oscillation frequency is 70 Hz ~120 Hz. The operation lasts 3–8 min until no visible solid residue left. Then, we performed centrifugation at 2000 rpm after the extraction of lipids in the tissue homogenate using chloroform and performed washing of extractions using chloroform/methanol mixture (2/1, v/v,) for twice. Last, we redissolve the evaporated solids using isopropanol/methanol mixture and collected supernatant after centrifugation at 12000 rpm.

We used chromatographic conditions as following: the column is Hypersil GOLD C18 (100 × 2.1 mm, 1.9 μm); the mobile phase A is acetonitrile: water (60:40, V/V), mobile Phase B is acetonitrile: isopropanol (10:90, V/V); the flow rate was 0.40 ml/min, the injection volume was 5 μl, and the column temperature was 45 °C. Lipids were separated via gradient elution under the following conditions: 0.0–4.0 min, 5% B; 4.0–25.0 min, 5–95% B; 25.0–30.0 min, 95% B; The flow rate was 0.4 mL/min. The MS ionization and data acquisition were performed using a Thermo Scientific™ Q Exactive™ hybrid quadrupole Orbitrap mass spectrometer with positive and negative model. The heated capillary temperature was 350 °C. The spray voltage was 3.0KV The Sheath Gas Flow rate, Aux Gas Flow Rate and Sweep Gas Flow Rate were 45 arb, 15 arb, 1arb. S-Lens RF Level was 50%. Scan ranges was 130–1950.

### Mass spectrometry data processing

High-throughput identification and relative quantification of lipids was performed separately for positive and negative ionization mode data, using LipidSearch software (Thermo Fisher Scientific) [31] and the default parameters for QExactive product search and alignment. “Comprehensive ID” was used as lipid identification module for untargeted methods, which discriminate each lipid by matching the predicted fragmentation pattern stored in the database. After alignment, row peak areas for each sample were normalized to percent of the total peak area, and the normalized matrix was used for further statistical analyses. Peaks were filtered according to the following predetermined quality control criteria: Rej (‘Reject’ parameter calculated by LipidSearch) equal to 0; PQ (‘Peak Quality’ parameter calculated by LipidSearch software) higher than 0.85; CV (standard deviation/mean peak area across triplicate injections of a represented (pooled) biological sample) below 0.3; ± ms deviation tolerance was 5 ppm.

Sample names, lipid information (species, subclass, structural information), and normalized peak areas were integrated and imported into SIMCA-P + 14.0 (Umetrics, Umeå, Sweden). Discrimination of lipid species between DL and HC samples was analyzed and visualized using partial least squares discriminant analysis (PLS-DA) [32]. To identify the differential lipid species responsible for discriminating between the two groups, we used threshold of variable importance plots (VIP) > 1.0 with statistical significance set at P < 0.05.

### Lipid reaction activities in lipid pathways

The reaction activities in lipid pathways can be predicted based on methods as described previously [33, 34]. This method calculated statistical *Z* scores for all possible lipid pathways in order to predict whether a particular pathway is active or inactive in DL as compared to HC. Reactions with higher *Z* scores were identified as active. First, we examined all possible lipid pathways from Reactome (http://www.reactome.org/) and constructed a pathway network. Based on the pathway network, we computed the reaction weight vector ($\omega _i$) as a ratio of product ($A_{i + 1}$) over substrate ($A_i$) for each edge in the network ($\omega _i = (A_{i + 1}/A_i)(i = 1,2, \ldots ,k - 1)$). Then, for each weighted edge of the pathway, one-sided Student’s *t* tests were performed using the weights between DL and HC, to identify reactions with differential activity. By assuming that the t-distribution can be approximated by a normal distribution, the *P* values were converted to *Z* scores using the cumulative distribution function *CDF*, *Z* = *CDF*^−1^(1−*P*). We chose the significance level (*P*) to be 0.05, corresponding to a *Z*_*i*_ > 1.645 for a reaction to be considered as significantly active. For visualization, the *Z* scores were multiplied by −1 for the reactions which were significantly more active in HC as opposed to DL. Finally, we calculated an average *Z* score for lipid pathways to detect consistent changes in the flux across multiple reaction steps. *Z* score for pathway A (*Z*_*A*_) was calculated by all $Z_i(i = 1,2, \ldots ,k - 1)$ of reactions in the pathway, as follows:

$$Z_A = \frac{1}{{\sqrt {k - 1} }}\mathop {\sum }\limits_{i = 1}^{k - 1} Z_i$$

As shown in references [33, 34], *Z*_*A*_ also follows a normal distribution. To determine if pathway A was significantly more active, we again chose the significance level (*P*) to be 0.05, corresponding to a *Z*_*i*_ > 1.645 for reaction to be considered as significantly active. Again, these *Z* scores were multiplied with −1 for the reactions which were significantly more active in HC as opposed to DL. For visualization, a negative *Z*-score indicates inactive pathway in DL relative to HC, and a positive *Z*-score indicates active pathway in DL relative to HC.

### Statistical analyses

Statistical analyses were carried out using SPSS (version 21) and R studio (version 3.5.2, 2018), unless otherwise described. The discriminating lipid species between the two groups were considered significant at the threshold of VIP > 1.0 and *P* values < 0.05. The levels of all lipid subclasses were analyzed by unpaired two-tailed Student’s *t* tests. Error bars represent the standard error of the mean (SEM) in all cases. LEfSe analysis was used to identify the different gut bacteria and viruses by estimating the effect of the abundance of each species (LDA > 2 and *P* value < 0.05). In established lipid pathways, the reactions were considered as significantly active (or inactive) for their calculated Z score >1.645 (or <−1.645), corresponding to the P-value < 0.05. Correlation data analysis was performed using the spearman’s regression model with the significance threshold of *P* < 0.05. The investigators were not blinded to the group classification while analyzing the data.

## Results

### Gut virus differences between DL and HC macaques

**Fig. 1:**
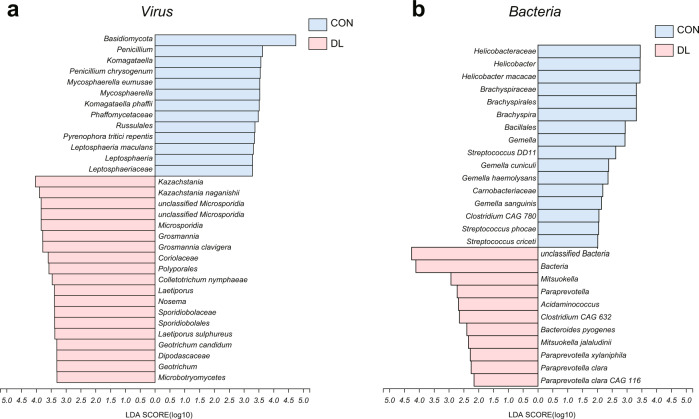
The virus and bacteria that discriminate DL from HC groups. LEfSe of bacteria and virus from phylum to species level. Linear discriminant analysis (LDA) combined with effect size measurements revealed a list of features that enable discrimination between the HC and DL groups in the fecal samples. **a** At the viral level, the DL subjects showed 16 enriched species, mainly belonging to families *Myoviridae* (7 species) and *Siphoviridae* (4 species), and 17 depleted species mainly from the families *Podoviridae* (6 species) and *Siphoviridae* (6 species). **b** At the bacterial level, the DL subjects showed 6 enriched species, mainly belonging to family *Paraprevotella* (3 species), and 8 depleted species mainly from the families *Streptococcaceae* (3 species) and *Gemella* (3 species). The discriminative variants (gut virus, bacteria species) were identified based on LDA score >2.5. Sample set: HC, *n* = 6; DL, *n* = 6.

The obtained metagenomic sequencing was mapped to the known viral genomes NCBI NR database. From the filtered sequence data, we annotated 1311 viral species based on the NR database. Using LEfSe analysis, we identified 33 differential gut viral species that could discriminate between the DL and HC groups (Fig. 1a, Supplementary Table 2). Compared to HCs, the DLs were characterized by 16 enriched gut viruses, mainly belonging to the *Myoviridae* (7 species) and *Siphoviridae* (4 species) families, and 17 depleted viruses mainly from the *Podoviridae* (6 species) and *Siphoviridae* (6 species) families. Interestingly, the majority of altered gut viruses (25/33, 75.76%) belonged to the order *Caudovirales*, suggesting altered *Caudovirales* ecology was a hallmark of the DL animals.

### Gut bacterial species differences between DL and HC macaques

Here, using our previously published metagenomic data [8], we reannotated 468 bacteria families and 11653 species based on NR database. Using LEfSe analysis, we identified 14 differential gut bacterial species responsible for distinguishing the DL and HC groups (Fig. 1b, Supplementary Table 3). Compared to the HC group, the DL group was characterized by 6 increased bacteria species, mainly belonging to the *Paraprevotella* family (3 species), and 8 decreased species mainly from the *Streptococcaceae* (3 species) and *Gemella* (3 species) families. The majority of altered bacterial species (9/14, 64.28%) belonged to the phylum Firmicutes.

### DL macaques have different lipid composition in depression relevant brain regions and plasma

**Fig. 2:**
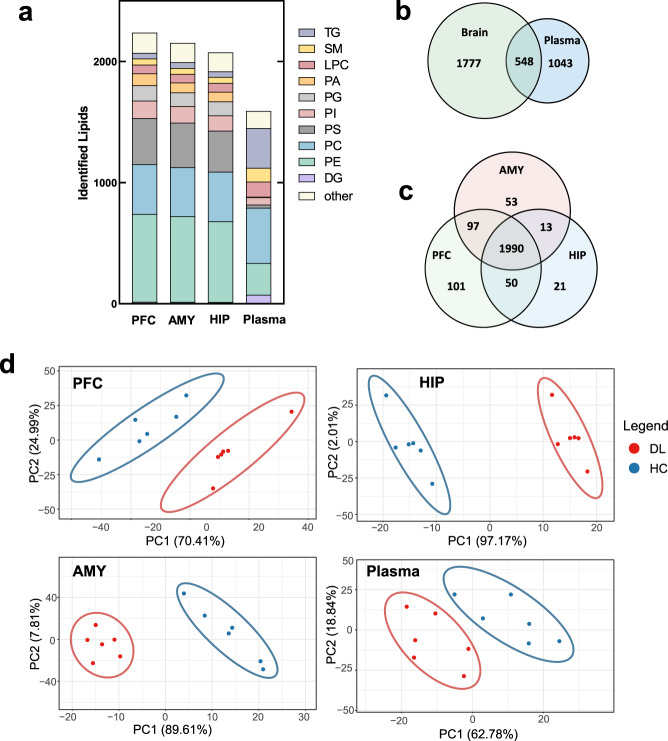
Profile of lipidomic changes in brain and plasma. **a** Composition of subclasses of total identified lipids in prefrontal cortex (PFC), amygdala (AMY), hippocampus (HIP) and plasma. Identified lipids exhibited similar composition of subclasses in 3 brain regions (2238, 2153, 2074, respectively). **b** Number of shared lipid species between 3 brain regions and plasma. **c** Number of shared and specific lipid species between 3 brain regions. **d** Partial Least Squares Discriminant Analysis (PLS-DA) showed that the PFC, HIP, AMY and plasma metabolic signatures of DL group were substantially different from that in the HC group (*n* = 6, HC, blue dots; *n* = 6, DL, red dots). PLS-DA was calculated by the relative abundance (normalized by total peak area). PC phosphatidylcholine, PE phosphatidylethanolamine, Cer ceramide, LPE lyso-Phosphatidylethanolamine, PA phosphatidic acids, PG phosphatidylglycerols, PI phosphatidylinositols, PS phosphatidylserines, SM sphingmyelin, DG 1,2-Diacylglycerol, LPC 2-Lysophosphatidylcholine, So sphingosine, TG Triacylglycerol.

In total, we identified 2238, 2153, and 2074 lipid species in PFC, AMY, and HIP, respectively. The proportions of lipid subtypes between brain regions were generally similar, with 1990 shared lipid species. Phosphatidylethanolamine (PE), PC, and phosphatidylserines (PS) were the most abundant species, together comprising 67.7%, 68.7%, and 68.2% of all lipid species in PFC, AMY, and HIP respectively (Fig. 2a, c). Plasma has low similarity with brain lipids, with 34.4% (548/1591) overlapped species. Lipid in plasma were mainly comprised of PE, PC, and triacylglycerol (TG) subtypes, together comprising 65.8% of the 1591 lipid species (Fig. 2a, b). PLS-DA was performed to determine whether the overall lipidomic signatures of DL macaques were significantly different from those of controls. We performed PLS-DA with the relative abundance (normalized by total peak area) of lipid species. These lipidomics assays identified robust differences between HCs and DLs in PFC, AMY, HIP, and plasma (Fig. 2d).

### DL macaques have different lipid levels and structure in depression relevant brain regions and plasma

**Fig. 3:**
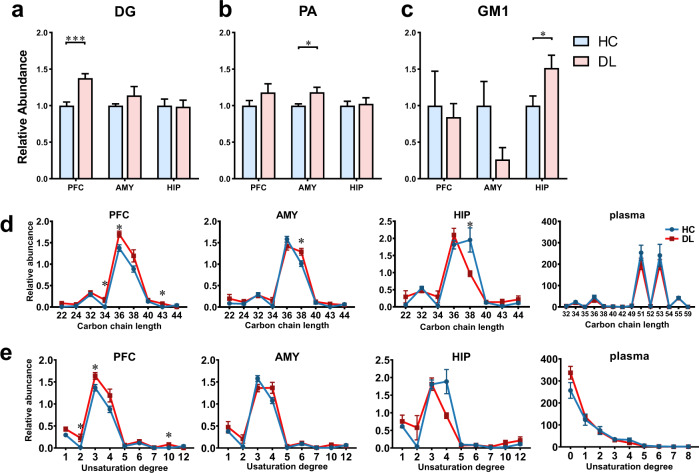
Significant changes exhibited in abundance and fatty acid composition of 1,2-Diacylglycerol (DG) in the PFC of DL macaques. **a–c** The abundance of some lipidomic subclasses showed significantly changes with specific brain regions, displaying the increased DG in PFC, increased PA in AMY and increased GM1 in HIP in the DL group relative to HC group. The abundance of lipidomic subclasses in DL (red bars) were normalized by whose relative abundance in HC group (blue bars). **d–e** Analysis of fatty acyl composition of DG species by total carbon chain length and total degree of unsaturation showed variation by brain region. The carbon chain length and unsaturation degree of DG ranged congruously in three brain regions, 34–40 and 2–5, respectively. The carbon chain length of DG altered in PFC, AMY and HIP; the unsaturation of DG altered in PFC *n* = 6 per group. *P < 0.05, **P < 0.01, ***P < 0.001, two-sided Student *t* test; bars and points show mean ± SEM.

We compared the relative abundance of each lipid subclass between DL and HC, and found that there were specifically altered lipid subclasses in the same three brain regions. Normalizing against the corresponding lipid level in HCs, we found DG was significantly increased in PFC (FC = 1.38, *P* = 0.0008) (Fig. 3a), PA was significantly increased in AMY (FC = 1.18, *P* = 0.04) (Fig. 3b), and monosialotetrahexosylganglioside (GM1) was significantly increased in HIP (FC = 1.51, *P* = 0.04) (Fig. 3c). The proportion of DG, PA and GM1 were similar between brain regions, with 66.7% (10/15) shared DG species, 65.7% (71/108) shared PA species and all shared GM1 species (Supplementary Fig. 1). Then we further identified the discriminating lipid species between DL and HC macaques using double cut-off (results were considered statistically significant if *P* < 0.05 and VIP > 1.0). Of the 72 total discriminating lipid species, we found that HIP had the most (28 species), while AMY had the least (7 species) (Supplementary Fig. 2). These results were consistent with what we previously reported based on untargeted metabonomics [8].

### DG lipid structure is altered in depression relevant brain regions of DL macaques

Brain function relies on the homeostasis of cellular membranes, and its perturbation might partially explain the neuronal deficits found in depression. The carbon chain length and the degree of unsaturation of fatty-acyls are two key elements of lipid architecture that impact membranes’ functional biophysical properties. Our lipidomic analyses demonstrated not only differences in relative lipid composition and levels between HC and DL macaques, but also differences in lipids’ biochemical structures with potential structure-function consequences on neural cell activity. Among the altered lipid subclasses, DG showed profound structural alterations, with multi-level alterations of carbon chain length and degree of unsaturation. Compared with HC, the DL macaques had altered levels of DGs with carbon chain lengths of 34C, 36C, and 43C in PFC, and 38C in AMY and HIP (Fig. 3d). The differential carbon chain length DGs were primarily in the range of 34C to 40C, a range which comprised the majority of DG species. We also analyzed the degree of unsaturation for each subclass and DG. Compared with HCs, the degree of unsaturation of DG was significantly increased in 2, 3, and 10 double bones in PFC, but there was no difference in unsaturation degree of DG in AMY or HIP (Fig. 3e). Taken together, DG structure and degree of unsaturation was the most different in the PFC, in HC versus DL macaques.

In the periphery (plasma), the structural changes in DG species were distinctly different than seen in brain. In plasma, the typical DG carbon chain length was longer, mainly 49C to 54C (Fig. 3d). The degree of unsaturation was lower, mainly 0 to 3 (Fig. 3e), compared with 2 to 6 carbon bones of DG in brain. There was no difference in carbon chain length or unsaturation degree in plasma DG.

### DG-related lipid pathway activity was altered in DL macaques

**Fig. 4:**
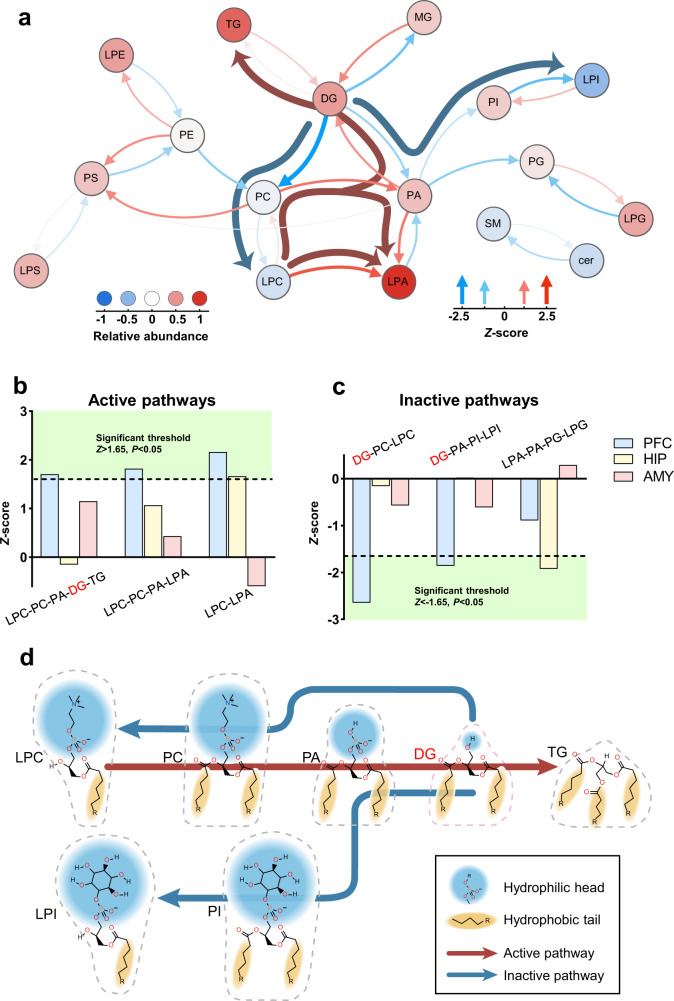
DG-related lipid pathway activity was particularly altered in the PFC of DL macaque. Analysis of lipid pathway activity showed altered predicted lipid fluxes occurred in brain regions. Calculated *Z*-scores were chosen to indicate the active/inactive pathways in DL relative to HC, with the positive/negative values. **a** The PFC of DL macaque showed major altered lipid pathways, which were 3 active pathways LPC-PC-PA-DG-TG, LPC-PC-PA-LPA and LPC-LPA, and 2 inactive pathway DG-PC-LPC, DG-PA-PI-LPI, respectively. Altered lipid pathway activity was calculated by *Z*-score of each lipid classes in DL (*n* = 6) relative to HC (*n* = 6). Red and blue arrows show reactions with positive and negative activity, respectively. Colored circles indicate the fold changes in lipid abundance between 2 groups (−0.47–1.0). Deep arrows indicate the shared lipid flux across the network. **b**, **c** The HIP of DL macaque showed minor altered lipid pathways, including active pathway LPC-LPA and inactive pathway LPA-PA-PG-LPG. **d** molecular geometric alteration of DG related lipid pathways. Blue and yellow backgrounds indicated head groups and hydrophobic part. Dashed frames showed the geometry of lipids molecule, which can be defined as cone (DG, PA, TG), cylinder (PC and PI) and inverted cone (LPI and LPC).

Given the altered abundance and structure of brain DG, next we employed lipid-reaction analyses to explore whether specific lipid pathways were dysregulated in key depression-relevant brain regions in the DL macaque. Based on the Reactome and KEGG databases, we measured each lipid reaction and mapped them into lipid pathways. Briefly, the upstream and downstream transformations of any lipid species can be viewed as an edge, that can be evaluated by statistical *Z*-scores for predicting active and inactive pathways. Using a significance level of *P* = 0.05, corresponding to *Z* > 1.645 for a reaction to be considered significantly activated, we found altered lipid pathway activity in PFC, HIP, and AMY. In the PFC of DL macaques, 3 lipid pathways were significantly activated (LPC-PC-PA-DG-TG, LPC-PC-PA-LPA, and LPC-LPA), and 2 lipid pathways significantly inactivated (DG-PC-LPC, DG-PA-PI-LPI) relative to HCs (Fig. 4a–c). In the HIP, the LPC-LPA pathway was activated, and the LPA-PA-PG-LPG pathway inactivated, in DL versus HC. (Fig. 4b, c, Supplementary Fig. 3a). LPC-LPA activity was significantly increased in both PFC and HIP (Fig. 4b), but otherwise no lipid pathway activity was significantly changed across more than one brain region examined (Fig. 4b, c). This most dysregulation of lipid pathway activity occurred in PFC (3 active, 2 inactive, in DL relative to HC), followed by HIP (one up, one down) (Fig. 4b, c).

We also calculated *Z*-scores for synthesis and degradation of DG and PA. Interestingly, for DL relative to HC, we found significantly activated synthesis and inactivated degradation of PA in the AMY (Supplementary Fig. 3c,d respectively), which corroborates the higher PA levels in DL AMY as measured by MS (Fig. 3b). However, the accumulation of DG in the DL PFC (Fig. 3a) appears to arise principally from inactivated degradation, although DG synthesis in the DL PFC trended toward an increase but was not significant (Supplementary Fig. 3c,d, respectively).

Next, we explored the molecular geometry of lipids in these altered pathways and found some interesting lipid shape changes in these altered lipid pathways in the PFC of the DL macaques. Based on the size ratio of the hydrophilic head to the hydrophobic tail, glycerophospholipids and glycerolipids can be classified into cone (small head, two or more hydrophobic tails; including DG, PA, and TG), cylinder (large head and two hydrophobic tails; including PC and PI), or inverted cone (large head and single hydrophobic tail; including LPI and LPC) shapes. As shown in Fig. 4f, the altered lipid pathways tend to promote (red arrow) lipid shape evolution from inverted cone lipids to cylinder and cone lipids, and to inhibit (blue arrows) the opposite evolution. DG lay at the intersection of these evolutions of lipid molecular geometry, and was involved in multiple processes.

### Co-occurrence network analysis of changes in gut viruses, bacteria, and DG levels in DL macaques versus HCs

**Fig. 5:**
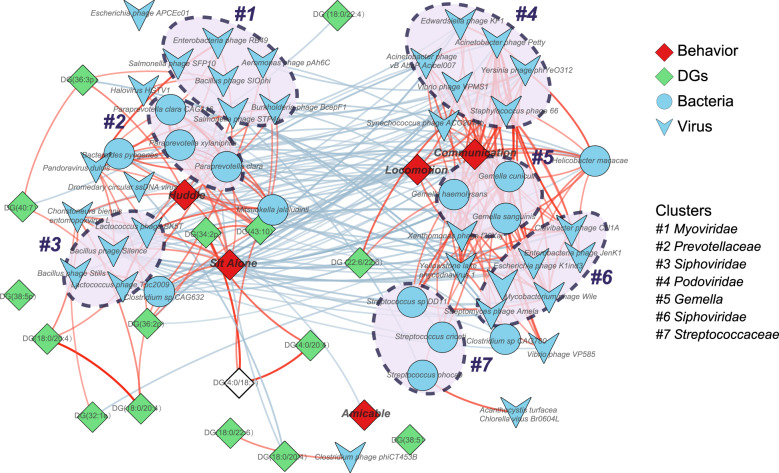
Network analyses of changed microbiome, DG in PFC and host phenotype interactions. The microbial species changed in DL were identified by LDA (LDA > 2), including14 bacterial and 33 viral species. Host-microbiota interaction network was built from Spearman’s non-parametric rank correlation coefficient (*P* < 0.05), and showed with coefficient >0.70 or <−0.70. Blue circles and V triangles indicate the altered bacteria and virus respectively, green rhombus indicate the DG species in PFC and red rhombus indicated the depressive behaviors. Edge-weighted Spring-Embedded Layout was used to cluster nodes in accordance with coefficient. In result, there were 3 clusters correlated with the behaviors that represent negative emotions and unhealthy effects (huddle and sit alone), mainly focused on bacterial family *Prevotellaceae* and viral families *Myoviridae* and *Siphoviridae*. There were 4 clusters correlated with behaviors that represent positive and healthy emotions (locomotion and communication), mainly focused on bacterial families *Gemella* and *Streptococcaceae* and viral families *Podoviridae* and *Siphoviridae*. No clusters correlated with amicable behavior that represents positive and healthy emotions. Red and blue lines indicate the correlation coefficient and the color depth was consistent with the coefficient (−0.70 – 0.70).

To explore the potential interactions of these microbiome and molecular changes along the MGB axis of DL macaques, we constructed co-occurrence networks of altered gut viruses, bacteria, and DG in the PFC of DL versus HC macaques. Using an edge-weighted spring-embedded layout, the network was visualized and the nodes were spontaneously mutually attractive or exclusive based on the coefficient between nodes (Fig. 5). Overall, co-occurrence analysis showed that gut viruses and bacteria formed strong and broad co-occurring relationships with DG levels in PFC; and the five behavioral phenotypes were divided, and the other nodes were spontaneously clustered with positive covariation around the behaviors. In the left region of this generated co-occurrence network, we found that two viral clusters (*Myoviridae* (#1), *Siphoviridae* (#3)) and a bacterial cluster (*Prevotellaceae* (#2)) were directly or indirectly substantially correlated with 11 DG species in PFC; meanwhile, those altered DG species in PFC of DL macaque were positively correlated with negative emotions behaviors (huddle and sit alone). In the right region of this network, alternations of two viral clusters (*Siphoviridae* (#6), *Podoviridae* (#4)) and two bacterial cluster (*Gemella* (#5), *Streptococcaceae* (#7)) were substantially correlated with communication and locomotion behaviors. Meanwhile, only one DG species in PFC was positively correlated with the two positive emotions behaviors. In addition, there were no gut viral or bacterial clusters distributed around the amicable behavior node. Together, our findings suggest that altered gut viral and bacterial species, and their interaction may be relevant to the onset of negative emotions behaviors by modulating the DG levels in PFC in the DL non-human primate model.

## Discussion

Growing evidence suggests that disturbed gut microbiome may contribute to depression pathology, but the specific mechanisms remain unclear. Here, we combined metagenomic and brain lipidomic analyses of *M. fascicularis* macaques with naturally occurring depression-like behaviors. We identified 33 altered viral species mainly belonging to *Myoviridae*, *Podoviridae*, and *Siphoviridae*, and found 14 altered bacterial species mainly belonging to *Paraprevotella*, *Streptococcaceae*, and *Gemella*. In the brain, we found marked disturbances of DG levels and structure in the PFC of DL macaques compared to controls. Moreover, lipid reaction networks identified more activated and inactivated lipid pathways in PFC than in AMY or HIP, with DG being a key nodal player in these PFC lipid pathways. Finally, co-occurrence analysis showed that altered gut viral and bacterial species, and their interaction were correlated with onset of negative emotions behaviors by modulating the DG levels in PFC.

Previously, the role of the gut virome has been unexplored in depression. However, importantly, we found that our DL animals had more altered gut viruses than gut bacteria, suggesting that the gut virome may play a role at least equivalent to that of the gut bacteriome in the pathology of depression. The three differential viral families were bacteriophages associated with gut bacteria, suggesting that such viruses may influence host behaviors via regulating their host bacteria. In the DL macaques, the altered phages mainly parasitize *Proteobacteria* and *Firmicutes* bacteria. Interestingly, we recently found similar viral disturbances in MDD patients, in which the altered gut viruses were mainly bacteriophages too. The MDD patients had increased *Siphoviridae* but decreased *Podoviridae* viral family populations, which aligns with our findings in the DL macaques described herein. These findings highlighted the potential role of gut viruses in depression, and emphasize that such phage disturbances may be both a hallmark and diagnostic of depression.

Recent clinical investigations have reported significant changes of gut microbiome in patients with MDD^36^, [35]. These clinical findings were partly inconsistent due to the demographic diversity of cohorts and analytical approaches. Our NHP depression-like model used herein provided an ideal model to avoid those confounding factors, due to the native social structure and habitat that are characteristic of this model. Like many other reports of the depression microbiome [23], we again identified disturbances of the phylum *Firmicutes*. The enriched microbiota in the DL macaques mainly belonged to the family *Paraprevotella*, and the depleted species mainly belonging to the genera *Gemella* and *Streptococcus*. *Paraprevotella* has been suggested as a biomarker in depression [36, 37] and attention deficit hyperactivity disorder, as well as a potential factor to inhibit plasma acetate levels and intrarenal RAS activation [38]. *Streptococcus* strains such as *Streptococcus salivarius* and *Streptococcus thermophilus*, previously recognized as pathogens, have recently been used as psychobiotics in mental health [39–41]. In our study, we also found some *Streptococcus* enriched in healthy controls, suggesting that gut *Streptococci* may play protective roles in the MGB axis.

Brain is particularly enriched in lipids, with a diverse lipid composition compared to other tissues [42]. Changes in the composition and structure of lipids in the brain profoundly affect neurodevelopment and signal transduction in perception and emotional behavior, which may lead to depression and anxiety disorders [43–45]. Previous lipidomic studies mainly focused on the amount of the different lipid species, which is reasonable but there are still some limitations: the variation of lipid structure and abundance. Recent studies have tried to scrutinize the structural and biotransformational alteration of lipids in disease. In our study, using a comprehensive approach, we tried to identify a key lipid group along the MGB axis that may be relevant to depression. DG in PFC was identified based on 4 aspects: first, the abundance of DG was significantly higher in PFC; second, the carbon chain length and unsaturation of DG were altered in at multiple levels; third, DG pathway activity was profoundly altered in PFC; last, DG in PFC was deeply involved in the MGB axis-behavior network.

In our previous studies and other preclinical and clinical experiments, disturbances of glycerolipid and glycerophospholipid metabolism were considered hallmarks in depression [46–49], but their role in depression pathogenesis is not explicitly clear. Here, using lipidomic approaches, we further showed alterations in DG pathway lipid shapes especially in the PFC of DL macaques. Both DG-related reaction pathways showed shape transformations from inverted cone and cylinder to cone via decreased headgroup size and more hydrophobic tails. Previous studies have reported that changes in bilayer curvature during vesicle fusion/fission relies on lipid shapes via CHOL translocations (chains) [14, 50, 51]. The cone shaped lipids, mainly PA and DG, promote negative membrane curvature via various phospholipases, while lyso-phosphatidylcholine (LPC) has only a single hydrocarbon tail that promotes positive membrane curvature [51]. Secondly, emerging evidences shows that the fatty acid chain length and unsaturation are involved in anxiety and cognitive disorders by modulating membrane fluidity [52, 53]. Here we found that the unsaturation degree of DG in PFC was altered, and the di- and tri-unsaturated DG significantly decreased, suggesting that the low unsaturated fatty acids may weaken the protection of poly-unsaturated fatty acids (PUFAs) in depression. As Levental et al. recently reported [54], exogenous PUFAs such as docosahexaenoic acid (DHA) and ω-6 arachidonic acid (AA) can reduce the di- and tri-unsaturated lipid species by counteracting cell membrane perturbations. Together, these findings may extend our understanding of brain lipids on depression.

Literature widely reported that gut microbiota can modulate hosts behaviors via the MGB-axis. Here we also found associations between gut microbiome and host DL behavioral phenotypes. Interestingly, virus and bacteria were spontaneously clustered, and those clusters as well as DGs surrounded different kinds of behaviors, suggesting the potential modulation between viruses, bacteria, and DL behaviors. Other studies have shown phages interfere with host bacteria. For example, Loeffler et al. found that *Podoviridae phage C1* can kill A, C, and E *streptococci* via lytic enzymes [55]. Romero et al. further confirmed that *Siphoviridae phiHER* can kill *Streptococcus pneumoniae* by specifically cleaving covalent bonds of cell wall peptidoglycan [56]. In line with these findings, in our results the *Podoviridae* (#4) and *Siphoviridae* (#6) virus clusters co-localized with the bacterial cluster *Streptococcaceae* (#7). These studies confirm that gut microbiota can modulate the host lipid metabolism in various ways, which may offer new therapeutic avenues for depression.

Interestingly, the five behavioral phenotypes of our DL macaque were spontaneously separated: huddle and sit alone, which can represent negative emotions and unhealthy affect in macaque, co-localized with the majority of DG species, and with 3 microbial clusters; locomotion and communication, which can represent positive emotions and healthy affect in macaque, were co-localized with only 1 species of DG and 4 microbial clusters; but amicable, another positive emotional behavior, located alone. These findings confirmed the reliability of our previously established behavior spectrum, and strongly suggested that the gut microbiota and brain lipids may modulate positive or negative emotion in different ways in depression. In our findings, DG mainly correlated with negative emotions rather than with positive emotions, suggesting that functional behavioral disturbances caused by changes in DG species composition, levels, and/or structure may serve to regulate or exacerbate negative emotions in depression. In contrast, positive emotions in depression may be modulated by the other unknown lipid groups. Interestingly, the unique DG species (DG (22:6/22:6)) that co-localized around positive emotions in our network analysis, is made up with a well-known fatty acid-docosahexaenoic acid (DHA). Many studies have investigated DHA supplementation as a potential treatment or prophylatic for depression. Van der Burg et al. found that DHA concentrations in red blood cell membranes were significantly correlated with a decrease in depressive symptoms during active treatment, and increased in response to depression treatment [57]. Weiser et al. fed pregnant rats with diets sufficient or deficient in DHA during gestation and lactation, and found that depressive-like behavior and its associated biomarkers in DHA-deficient offspring were worse compared with animals with sufficient levels of DHA [58]. In both these studies, the fatty acids were ingested, meaning that gut microbes would have participated in the absorption of these dietary fatty acid supplements. Based on our and these results, further studies of the intertwined roles of the gut microbiome and lipids in the pathology and pathogenesis of depression are warranted.

Nonetheless, there are some limitations of our study, which can also provide direction for future research. First, due to the low reproductive rate and morbidity, as well as ethical considerations, the sample sizes were relatively small, thus the reliability of the association reported may be impacted. Second, the effects of brain lipids that we reported in this study need further longitudinal independent validations with larger samples. The key viruses and bacteria need further isolation and culturing from fecal samples, and more independent verifications in multiple animal models, such as fecal microbiota transplantation (FMT) or microbial agents. Third, we used untargeted lipidomics and relative peak area normalization, which would not convey the true abundance of brain lipids. Further studies based on the targeted lipidomics methods is required. The 72 discriminating lipid species can be seen as promising entry point. Fourth, MGB crosstalk in depression involves multiple mechanisms and metabolic pathways beyond those which we focused on in this study. The vagus nerve, hypothalamic–pituitary–adrenal axis, and neuroimmune mechanisms, are all worthy of further study.

## Conclusions

Taken together, using metagenomic data, we found the altered gut virome, especially bacteriophages, plays a role in the onset of the DL macaque. Through multiomics approaches, we have presented evidence that DL macaques were characterized by disturbances of gut-virus, bacteria, and DGs in the PFC. Moreover, we found that disturbances of gut microbiome may be relevant to the onset of negative emotions behaviors by modulating the DG levels in the DL model. Our findings provide new directions to uncover the pathogenesis of depression.

## Declarations

### Ethics approval and consent to participate

This study was performed in strict accordance with the recommendations in the “Guide for the Care and Use of Laboratory Animals” of the Institute of Neuroscience at Chongqing Medical University (#20100031). All work involving NHPs was conducted in accordance with the NIH guide for the care and use of laboratory animals (https://www.ncbi.nlm.nih.gov/books/NBK54050/) and with the recommendations of the Weatherall report, “The use of non-human primates in research” [59]. We also followed nc3r recommendations (https://www.nc3rs.org.uk/) by using the minimum number of depressed macaques and age-matched controls, while maintaining statistical reliability. The sample sizes (*n* = 6/every group) were chosen based on the data of previous NHP studies [17, 18], and our previous works on behavior and biochemical measures in *M. fascicularis* [19]. The *M. fascicularis* facilities, housing, and primate laboratories used in this study are accredited by the Association for Assessment and Accreditation of Laboratory Animal Care. Macaques were housed in an environmentally controlled facility (22 ± 1 °C temperature; 50 ± 5% relative humidity; and 12 h light/12 h dark cycle with lights on at 7:00 AM). This study does not involve the use of human subjects.

### Statement about randomization and blinding

Randomized methods were not used in allocating sample to experimental groups. Blinded methods were used in evaluation of behavior phenotypes.

## Supplementary information

Supplementary Figure 1

Supplementary Figure 2

Supplementary Figure 3

Supplementary Table 1

Supplementary Table 2

Supplementary Table 3
