# Supplementary material for: Changes in gut viral and bacterial species correlate with altered 1,2-diacylglyceride levels and structure in the prefrontal cortex in a depression-like non-human primate model
Source: Transl Psychiatry. 2022 Feb 22;12:74. doi: 10.1038/s41398-022-01836-x (PMC8863841; doi:10.1038/s41398-022-01836-x)
Supplement: Supplementary file 1 — Supplementary Figure 1 [file 41398_2022_1836_MOESM1_ESM.docx]

**Supplementary Figure 1. Venn diagram showing the similarity of species composition**

**
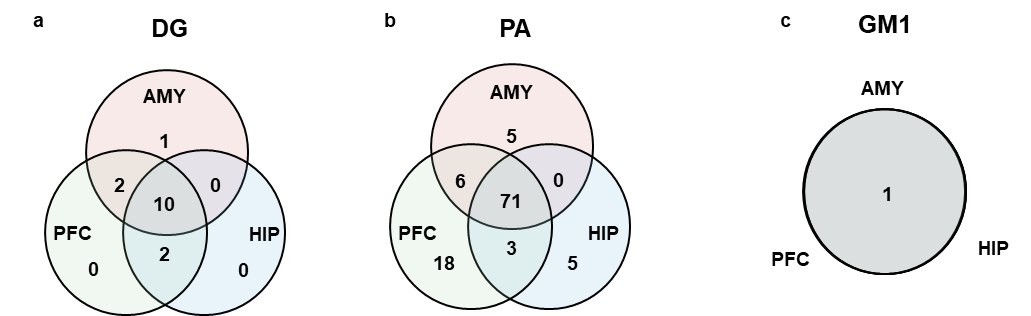
**

**Supplementary Figure 1. Venn diagram showing the similarity of species composition**

**(a).** 10 species of DG were overlapped between DG, PFC and HIP(76.9%, 71.4% and 83.3%, respectively). **(b).** 71 species of PA were overlapped between DG, PFC and HIP(86.6%, 72.4% and 89.9%, respectively). **(c).** all of GM1 were shared between 3 brain regions.
