# Supplementary material for: Changes in gut viral and bacterial species correlate with altered 1,2-diacylglyceride levels and structure in the prefrontal cortex in a depression-like non-human primate model
Source: Transl Psychiatry. 2022 Feb 22;12:74. doi: 10.1038/s41398-022-01836-x (PMC8863841; doi:10.1038/s41398-022-01836-x)
Supplement: Supplementary file 2 — Supplementary Figure 2 [file 41398_2022_1836_MOESM2_ESM.docx]

**Supplementary Figure 2. Discriminating lipids in brain regions and plasma between HC and DL groups.**

**
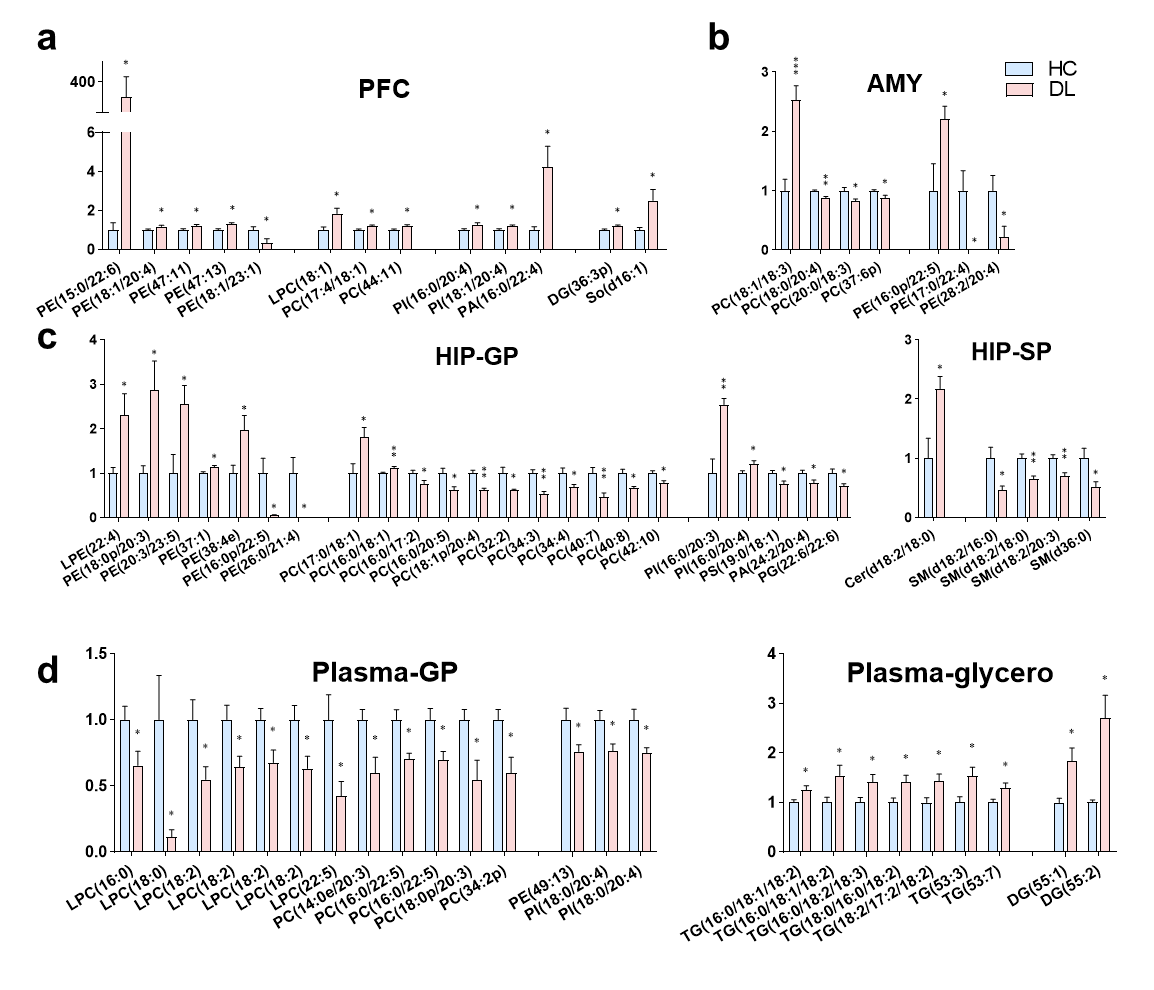
**

**Supplementary Figure 2. Discriminating lipids in brain regions and plasma between HC and DL groups.** Lipidomic analyses were performed on three brain regions and plasma of HC (blue bars) and DL(red bars) groups. Y values expressed as relative levels to control animals. Totally, we identified 13, 7, 28 and 24 discriminating lipids between two groups in PFC, AMY, HIP and plasma, respectively. **(a)** most (91%, 10/11) of glycerophospholipids(GP) were increased in PFC; **(b)**71% (5/7) of glycerophospholipids(GP) were decreased in AMY; **(c)**most(81%, 9/11) of PC and all SM were decreased in HIP; **(d)**all of GPs(n=12, mainly LPC and PC) were increased and all of glycerolipids (n=9, mainly TG and DG) were decreased in plasma. n=6 per group. Discriminating lipids were identified on SIMCA, with the significance threshold of variable importance plot(VIP)>1.0 P-values < 0.05. *P < 0.05, ** P < 0.01, ***P < 0.001, two-sided Student T-test; bars show mean ± SEM.
