# Supplementary material for: Changes in gut viral and bacterial species correlate with altered 1,2-diacylglyceride levels and structure in the prefrontal cortex in a depression-like non-human primate model
Source: Transl Psychiatry. 2022 Feb 22;12:74. doi: 10.1038/s41398-022-01836-x (PMC8863841; doi:10.1038/s41398-022-01836-x)
Supplement: Supplementary file 3 — Supplementary Figure 3 [file 41398_2022_1836_MOESM3_ESM.docx]

**Supplementary Figure 3. Pathway activity of lipid showed changed reactions among brain regions.**


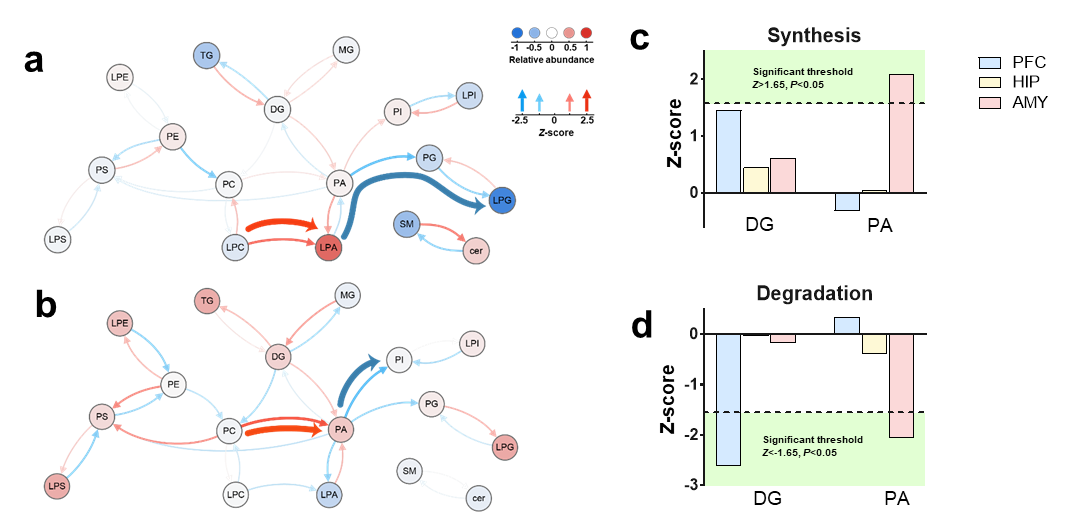


**Supplementary Figure 3. Pathway activity of lipid showed changed reactions among brain regions** **in DL group relative to HC group.** Analysis of lipid pathway activity showed altered predicted lipid fluxes occurred in three brain regions. **(a)** HIP showed an active lipid pathway LPC-LPA, and inactive pathway LPA-PA-PG-LPG. **(b)** AMY showed an active lipid pathway PC-PA, and inactive pathway PA-PI. **(c-d)** Predicted synthesis and degradation showed the significantly active synthesis and inactive degradation of PA in AMY, as well as significantly inactive degradation of DG in PFC. Pathway activity was predicted on the basis of lipidomics data. One-sided Student’s t tests (forward or reverse reaction) were used to calculate P values before z transformation. Dashed line indicates significance threshold (*Z*=1.65, P=0.05).
