# Supplementary material for: Changes in gut viral and bacterial species correlate with altered 1,2-diacylglyceride levels and structure in the prefrontal cortex in a depression-like non-human primate model
Source: Transl Psychiatry. 2022 Feb 22;12:74. doi: 10.1038/s41398-022-01836-x (PMC8863841; doi:10.1038/s41398-022-01836-x)
Supplement: Supplementary file 4 — Supplementary Table 1 [file 41398_2022_1836_MOESM4_ESM.docx]

**Supplementary Table 1. Depressive phenotypes of enrolled Macaca fascicularis samples**

| **Behavior** | **DEP** | | **HC** | | **P-value** |
| --- | --- | --- | --- | --- | --- |
|  | mean±SD | n | mean±SD | n |  |
| **Huddle** | 196.22±255.32 | 110 | 0.60±2.90 | 110 | 5.85E-14 |
| **Locomotion** | 75.47±89.43 | 110 | 418.36±243.08 | 110 | 7.97E-32 |
| **Ingestion** | 40.76±99.19 | 110 | 57.22±96.18 | 110 | 0.21 |
| **Sit alone** | 917.85±273.79 | 110 | 26.05±32.15 | 110 | 6.11E-89 |
| **Amicable** | 92.21±273.79 | 110 | 139.89±128.71 | 110 | 2.85E-02 |
| **Communication with male** | 28.14±15.09 | 6 | 381.94±195.3 | 6 | 1.29E-03 |

Note: 1. The behavioral data for each monkey was the average value from 18 observations in 3 days, n indicated the repeats of observations.

2. The behavioral test data has been previously published^1^.

**Reference**

1. Zheng P, et al. The gut microbiome modulates gut-brain axis glycerophospholipid metabolism in a region-specific manner in a nonhuman primate model of depression. *Mol Psychiatry*(2020).
