# Supplementary material for: Changes in gut viral and bacterial species correlate with altered 1,2-diacylglyceride levels and structure in the prefrontal cortex in a depression-like non-human primate model
Source: Transl Psychiatry. 2022 Feb 22;12:74. doi: 10.1038/s41398-022-01836-x (PMC8863841; doi:10.1038/s41398-022-01836-x)
Supplement: Supplementary file 5 — Supplementary Table 2 [file 41398_2022_1836_MOESM5_ESM.docx]

**Supplementary Table 2. Discriminatory gut virus between DL from HC group**

| **Family** | **Genus** | **Species** | **DL** | | **HC** | | **Fold change**  **(DL/HC)** | **Enrichment** | **LDA** | **p value** |
| --- | --- | --- | --- | --- | --- | --- | --- | --- | --- | --- |
|  |  |  | **mean** | **sem** | **mean** | **sem** |  |  |  |  |
| *Circoviridae* | *unclassified Circoviridae* | *Dromedary stool-associated circular ssDNA virus* | 1.72E-05 | 2.44E-05 | 3.23E-06 | 4.22E-06 | 5.306 | up | 2.461 | 0.0278 |
| *Myoviridae* | *Pbunalikevirus* | *Burkholderia phage BcepF1* | 9.04E-06 | 7.64E-06 | 5.40E-07 | 1.32E-06 | 16.748 | up | 2.924 | 0.0132 |
| *Myoviridae* | *unclassified Myoviridae* | *Bacillus phage SIOphi* | 2.10E-05 | 1.80E-05 | 1.63E-06 | 2.93E-06 | 12.845 | up | 2.454 | 0.0201 |
| *Myoviridae* | *unclassified Myoviridae* | *Aeromonas phage pAh6-C* | 3.00E-06 | 2.33E-06 | 0.00E+00 | 0.00E+00 | - | up | 2.945 | 0.0222 |
| *Myoviridae* | *unclassified Myoviridae* | *Vibrio phage VP58.5* | 0.00E+00 | 0.00E+00 | 1.88E-05 | 3.13E-05 | 0.000 | down | 2.461 | 0.0222 |
| *Myoviridae* | *T4likevirus* | *Synechococcus phage ACG-2014b* | 5.17E-07 | 1.27E-06 | 3.69E-05 | 7.26E-05 | 0.014 | down | 2.391 | 0.0326 |
| *Myoviridae* | *unclassified Myoviridae* | *Escherichia phage APCEc01* | 2.55E-05 | 1.75E-05 | 1.41E-05 | 2.08E-05 | 1.810 | up | 2.279 | 0.0374 |
| *Myoviridae* | *T4likevirus* | *Salmonella phage STP4-a* | 5.82E-05 | 4.76E-05 | 9.54E-06 | 8.87E-06 | 6.101 | up | 2.212 | 0.0450 |
| *Myoviridae* | *T4likevirus* | *Enterobacteria phage RB49* | 5.97E-06 | 5.91E-06 | 8.37E-07 | 2.05E-06 | 7.126 | up | 2.667 | 0.0495 |
| *Myoviridae* | *Viunalikevirus* | *Salmonella phage SFP10* | 4.56E-06 | 3.95E-06 | 6.16E-07 | 1.51E-06 | 7.405 | up | 3.130 | 0.0495 |
| *Phycodnaviridae* | *unclassified Phycodnaviridae* | *Yellowstone lake phycodnavirus 1* | 2.35E-07 | 5.76E-07 | 2.14E-05 | 2.07E-05 | 0.011 | down | 2.037 | 0.0210 |
| *Phycodnaviridae* | *Chlorovirus* | *Acanthocystis turfacea Chlorella virus Br0604L* | 4.62E-07 | 1.13E-06 | 8.16E-05 | 1.41E-04 | 0.006 | down | 2.450 | 0.0495 |
| *Podoviridae* | *unclassified Podoviridae* | *Vibrio phage VPMS1* | 1.45E-06 | 1.19E-06 | 4.04E-03 | 9.34E-03 | 0.000 | down | 3.183 | 0.0064 |
| *Podoviridae* | *Phikmvlikevirus* | *Acinetobacter phage Petty* | 0.00E+00 | 0.00E+00 | 1.69E-05 | 2.22E-05 | 0.000 | down | 2.350 | 0.0074 |
| *Podoviridae* | *T7likevirus* | *Yersinia phage phiYeO3-12* | 0.00E+00 | 0.00E+00 | 7.43E-06 | 7.26E-06 | 0.000 | down | 3.020 | 0.0074 |
| *Podoviridae* | *unclassified Podoviridae* | *Acinetobacter phage vB AbaP Acibel007* | 1.31E-06 | 3.20E-06 | 1.18E-05 | 1.79E-05 | 0.110 | down | 3.270 | 0.0326 |
| *Podoviridae* | *unclassified Podoviridae* | *Edwardsiella phage KF-1* | 5.08E-06 | 1.25E-05 | 1.70E-03 | 3.84E-03 | 0.003 | down | 2.977 | 0.0326 |
| *Podoviridae* | *Ahjdlikevirus* | *Staphylococcus phage 66* | 3.53E-05 | 2.63E-05 | 6.61E-03 | 1.47E-02 | 0.005 | down | 3.425 | 0.0374 |
| *Poxviridae* | *Betaentomopoxvirus* | *Choristoneura biennis entomopoxvirus 'L'* | 2.08E-05 | 1.59E-05 | 5.50E-06 | 2.68E-06 | 3.779 | up | 2.374 | 0.0374 |
| *Siphoviridae* | *unclassified Siphoviridae* | *Bacillus phage Stills* | 6.13E-05 | 1.13E-04 | 6.13E-07 | 1.50E-06 | 100.147 | up | 2.527 | 0.0132 |
| *Siphoviridae* | *unclassified Siphoviridae* | *Bacillus phage Silence* | 2.56E-05 | 2.20E-05 | 2.42E-06 | 5.93E-06 | 10.591 | up | 2.269 | 0.0201 |
| *Siphoviridae* | *unclassified Siphoviridae* | *Streptomyces phage Amela* | 3.02E-06 | 7.41E-06 | 6.28E-05 | 1.11E-04 | 0.048 | down | 2.132 | 0.0201 |
| *Siphoviridae* | *unclassified Siphoviridae* | *Enterobacteria phage JenK1* | 0.00E+00 | 0.00E+00 | 8.89E-06 | 9.67E-06 | 0.000 | down | 2.643 | 0.0222 |
| *Siphoviridae* | *unclassified Siphoviridae* | *Escherichia phage K1-ind(3)* | 0.00E+00 | 0.00E+00 | 3.86E-06 | 3.11E-06 | 0.000 | down | 2.796 | 0.0222 |
| *Siphoviridae* | *unclassified Siphoviridae* | *Mycobacterium phage Wile* | 0.00E+00 | 0.00E+00 | 7.99E-06 | 1.10E-05 | 0.000 | down | 2.681 | 0.0222 |
| *Siphoviridae* | *unclassified Siphoviridae* | *Lactococcus phage Tuc2009* | 3.22E-05 | 3.56E-05 | 3.71E-06 | 4.95E-06 | 8.666 | up | 2.605 | 0.0240 |
| *Siphoviridae* | *unclassified Siphoviridae* | *Enterococcus phage vB IME199* | 1.89E-03 | 2.67E-03 | 2.03E-02 | 2.66E-02 | 0.093 | down | 3.787 | 0.0374 |
| *Siphoviridae* | *unclassified Siphoviridae* | *Lactococcus phage BK5-T* | 2.64E-05 | 2.24E-05 | 9.59E-06 | 6.38E-06 | 2.751 | up | 2.388 | 0.0374 |
| *Siphoviridae* | *unclassified Siphoviridae* | *Clavibacter phage CN1A* | 2.20E-07 | 5.39E-07 | 1.34E-05 | 2.33E-05 | 0.016 | down | 2.616 | 0.0493 |
| *unclassified Viruses* | *unclassified Viruses* | *Pandoravirus dulcis* | 7.76E-06 | 7.88E-06 | 0.00E+00 | 0.00E+00 | - | up | 2.484 | 0.0222 |
| *unclassified Viruses* | *unclassified Viruses* | *Xanthomonas phage f20-Xaj* | 0.00E+00 | 0.00E+00 | 1.38E-05 | 2.73E-05 | 0.000 | down | 2.579 | 0.0222 |
| *unclassified Viruses* | *unclassified Viruses* | *Clostridium phage phiCT453B* | 1.01E-05 | 5.42E-06 | 5.06E-06 | 4.98E-06 | 1.988 | up | 2.709 | 0.0370 |
| *unclassified Viruses* | *unclassified Viruses* | *Halovirus HCTV-1* | 1.58E-05 | 3.35E-05 | 6.82E-06 | 9.21E-06 | 2.317 | up | 2.737 | 0.0495 |
