# Supplementary material for: Changes in gut viral and bacterial species correlate with altered 1,2-diacylglyceride levels and structure in the prefrontal cortex in a depression-like non-human primate model
Source: Transl Psychiatry. 2022 Feb 22;12:74. doi: 10.1038/s41398-022-01836-x (PMC8863841; doi:10.1038/s41398-022-01836-x)
Supplement: Supplementary file 6 — Supplementary Table 3 [file 41398_2022_1836_MOESM6_ESM.docx]

**Supplementary Table 3.** **Discriminatory bacterial species between DL from HC group.**

| **Family** | **Genus** | **Species** | **DL** | | **HC** | | **Fold change**  **(DL/HC)** | **Enrichment** | **LDA** | **p value** |
| --- | --- | --- | --- | --- | --- | --- | --- | --- | --- | --- |
|  |  |  | **mean** | **sem** | **mean** | **sem** |  |  |  |  |
| *Bacteroidaceae* | *Bacteroides* | *Bacteroides pyogenes* | 1.40E-01 | 3.11E-02 | 9.26E-02 | 2.46E-02 | 1.514 | up | 2.39 | 0.025 |
| *Clostridiaceae* | *Clostridium* | *Clostridium sp. CAG:780* | 1.06E-02 | 6.56E-03 | 3.06E-02 | 2.27E-02 | 0.348 | down | 2.05 | 1.04E-02 |
| *Clostridiaceae* | *Clostridium* | *Clostridium sp. CAG:632* | 1.32E-01 | 1.02E-01 | 5.07E-02 | 1.58E-02 | 2.592 | up | 2.64 | 0.037 |
| *unclassified Bacillales* | *Gemella* | *Gemella cuniculi* | 6.04E-04 | 1.41E-04 | 4.31E-02 | 9.65E-02 | 0.014 | down | 2.38 | 3.95E-03 |
| *unclassified Bacillales* | *Gemella* | *Gemella sanguinis* | 1.13E-03 | 1.85E-04 | 2.54E-02 | 5.22E-02 | 0.045 | down | 2.13 | 3.95E-03 |
| *unclassified Bacillales* | *Gemella* | *Gemella haemolysans* | 2.07E-03 | 6.43E-04 | 4.24E-02 | 8.98E-02 | 0.049 | down | 2.35 | 0.025 |
| *Helicobacteraceae* | *Helicobacter* | *Helicobacter macacae* | 2.22E-02 | 3.08E-02 | 5.14E-01 | 5.43E-01 | 0.043 | down | 3.43 | 1.04E-02 |
| *Veillonellaceae* | *Mitsuokella* | *Mitsuokella jalaludinii* | 5.83E-02 | 4.39E-02 | 1.69E-02 | 6.98E-03 | 3.451 | up | 2.34 | 0.025 |
| *Prevotellaceae* | *Paraprevotella* | *Paraprevotella clara* | 1.21E-01 | 2.59E-02 | 8.38E-02 | 2.03E-02 | 1.449 | up | 2.25 | 1.04E-02 |
| *Prevotellaceae* | *Paraprevotella* | *Paraprevotella xylaniphila* | 1.25E-01 | 2.51E-02 | 8.58E-02 | 1.98E-02 | 1.461 | up | 2.28 | 1.04E-02 |
| *Prevotellaceae* | *Paraprevotella* | *Paraprevotella clara CAG:116* | 9.03E-02 | 2.19E-02 | 6.18E-02 | 1.31E-02 | 1.461 | up | 2.15 | 0.016 |
| *Streptococcaceae* | *Streptococcus* | *Streptococcus phocae* | 1.48E-04 | 2.71E-05 | 1.91E-02 | 4.62E-02 | 0.008 | down | 2.03 | 1.04E-02 |
| *Streptococcaceae* | *Streptococcus* | *Streptococcus criceti* | 5.10E-04 | 3.36E-04 | 1.84E-02 | 4.27E-02 | 0.028 | down | 2.01 | 0.037 |
| *Streptococcaceae* | *Streptococcus* | *Streptococcus sp. DD11* | 1.98E-03 | 1.57E-03 | 7.49E-02 | 1.72E-01 | 0.026 | down | 2.61 | 0.037 |
